# Role of nebulized colistin as a substitutive strategy against nosocomial pneumonia caused by CR-GNB in intensive care units: a retrospective cohort study

**DOI:** 10.1186/s13613-022-01088-4

**Published:** 2023-01-07

**Authors:** Jia-Yih Feng, Jhong-Ru Huang, Chang-Ching Lee, Yen-Han Tseng, Sheng-Wei Pan, Yuh-Min Chen, Kuang-Yao Yang

**Affiliations:** 1 Department of Chest Medicine, Taipei Veterans General Hospital, #201, Sec. 2, Shih-Pai Road, Taipei, 11217 Taiwan; 2 School of Medicine, National Yang Ming Chiao Tung University, Taipei, Taiwan; 3 Institute of Public Health, National Yang Ming Chiao Tung University, Taipei, Taiwan; 4 Institute of Emergency and Critical Care Medicine, School of Medicine, National Yang Ming Chiao Tung University, Taipei, Taiwan; 5 Cancer Progression Research Center, National Yang Ming Chiao Tung University, Taipei, Taiwan

**Keywords:** Nosocomial pneumonia, Colistin, CR-GNB, Clinical failure, Mortality

## Abstract

**Background:**

Adverse reactions, especially nephrotoxicity, are great concerns of intravenous colistin treatment. The role of substitutive nebulized colistin in treating nosocomial pneumonia caused by carbapenem-resistant Gram-negative bacterial (CR-GNB) in critically ill patients remains unknown.

**Methods:**

This retrospective study enrolled patients with nosocomial pneumonia caused by colistin-susceptible CRGNB in the intensive care unit (ICU) without intravenous colistin treatment. Patients were categorized based on whether substitutive nebulized colistin was used alongside other intravenous antibiotics. Clinical responses and mortality rates were compared between the two groups in the original and propensity score (PS)-matched cohorts. This study aimed to investigate the clinical effectiveness of substitutive nebulized colistin in treatment outcomes of nosocomial pneumonia caused by CR-GNB. The impact of dosing strategy of nebulized colistin was also explored.

**Results:**

In total, 343 and 214 patients with and without substitutive nebulized colistin, respectively, were enrolled for analysis. In the PS-matched cohort, clinical failure rates on day 7 (22.6 vs. 42.6%, *p* = 0.001), day 14 (27.0 vs. 42.6%, *p* = 0.013), and day 28 (27.8 vs. 41.7%, *p* = 0.027) were significantly lower in patients with nebulized colistin. In multivariate analysis, nebulized colistin was an independent factor associated with lower day 14 clinical failure (Original cohort: adjusted odds ratio (aOR) 0.45, 95% confidence interval (CI) 0.30–0.67; PS-matched cohort: aOR 0.48, 95% CI 0.27–0.87). There were no differences in clinical failure rate and mortality rate between patients receiving high (> 6 MIU/day) and low (≤ 6 MIU/day) dose nebulized colistin in the PS-matched cohort.

**Conclusions:**

In ICU-admitted patients with nosocomial pneumonia caused by colistin-susceptible CRGNB, substitutive nebulized colistin was associated with better clinical outcomes.

**Supplementary Information:**

The online version contains supplementary material available at 10.1186/s13613-022-01088-4.

## Background

Nosocomial pneumonia, including hospital-acquired pneumonia (HAP) and ventilator-associated pneumonia (VAP), are the leading causes of morbidity and mortality in the intensive care unit (ICU) [1, 2]. Nosocomial pneumonia caused by carbapenem-resistant Gram-negative bacteria (CR-GNB) is a growing concern worldwide due to limited treatment options and poor prognosis [3–6]. VAP-prevention strategies have recently been grouped together into care bundles. These strategies, including hand hygiene, daily sedation interruption, protocolized weaning evaluation, and semirecumbent positioning, have been shown to effectively reduce rates of VAP in ICU in Taiwan and other areas [7–9]. Although a few novel antibiotics against CR-GNB were developed in recent years, their activity varied in GNB with differing resistance mechanisms [10]. Moreover, some novel agents are not available in many countries. Combination therapy is generally recommended for CR-GNB-related infection with moderate-to-high severity [11–13]. However, the optimal combination regimen remains unknown.

Colistin is effective against many GNBs and poses a high sensitivity rate against CR-*Acinetobacter baumannii* complex (CRAB), CR-enterobacteriaceae (CRE), and CR-*Pseudomonas aeroginosa* (CRPA). Colistin exerts its bactericidal effects by disrupting the integrity of the outer membrane, resulting in leakage of the cytoplasmic content, and neutralization of GNB endotoxins [14]. In the past, a before–after prospective study suggested that the daily intratracheal administration of colistimethate sodium at a dose of 1.6 million international units during 2 weeks, could significantly reduce the incidence of Gram-negative nosocomial bronchopneumonia in a population of critically ill patients on mechanical ventilation [15]. Despite its increasing use for managing nosocomial infection, administering intravenous (IV) colistin is frequently limited by its adverse reactions, especially nephrotoxicity [16]. Meanwhile, the low penetration of colistin in lung tissue may lead to inadequate antibacterial activity of intravenous colistin [17, 18].

Nebulization has been shown to increase colistin concentration in lung tissue effectively [17]. The role of adjunctive nebulized colistin remains controversial in most recent HAP/VAP guidelines and CR-GNB treatment guidance due to limited evidences from randomized clinical trials [4, 19]. Nebulized colistin in substitutive strategy, which means nebulized colistin without intravenous colistin [20], has also been used occasionally, especially in patients with impaired renal function and concerns of nephrotoxicity related to intravenous colistin. However, clinical evidences regarding the effectiveness of substitutive nebulized colistin are more scarce. This retrospective study included critically ill patients with nosocomial pneumonia caused by CR-GNB. We aimed to investigate the impact of substitutive nebulized colistin in the treatment outcomes of these patients. The differences in treatment outcomes between high- and low-dose substitutive nebulized colistin was analyzed as well.

## Materials and methods

### Patients and settings

This is a retrospective cohort study conducted in a referral medical center in Taiwan. From January 2016 to December 2019, ICU-admitted patients with nosocomial pneumonia caused by colistin-susceptible CR-GNB and did not receive intravenous colistin were eligible for enrollment. The inclusion criteria included: (a) ICU-admitted patients diagnosed with nosocomial pneumonia developing more than 48 h after admission. (b) Positive cultures of CR-GNB isolated from respiratory specimens, including sputum, tracheal aspirates, and bronchoalveolar lavage fluid. Exclusion criteria included: age < 20 years, diagnosis of community acquired pneumonia or health-care associated pneumonia, concomitant lung cancer with obstructive pneumonitis, CR-GNB that were resistant to colistin, did not receive anti-CRGNB antibiotics (including sulbactam, tigecycline, and carbapenem), and received intravenous colistin. The study protocol was approved by the Institutional Review Board of Taipei Veterans General Hospital and informed consent form was waived. (2020-11-006AC).

### Diagnosis of pneumonia and microbiological tests

The diagnosis of pneumonia was based on new or progressive infiltrates in the chest radiograph, in addition to at least two clinical findings suggestive of pneumonia. The suggestive clinical findings included exacerbated cough, increased production of purulent sputum, fever (≥ 38 °C) or hypothermia (< 35 °C), and leukocytosis (white cell count ≥ 10,000/cumm) or leukopenia (white cell count < 4000/cumm). HAP was defined as pneumonia occurring ≥ 48 h after hospital admission. Whereas, VAP was defined as pneumonia developing ≥ 48 h after endotracheal intubation with invasive mechanical ventilator. Causative organisms were defined as CR-GNB isolated from respiratory specimens, including sputum, endotracheal aspirates (moderate or heavy growth by semiquantitative method), and bronchoalveolar lavage fluid with a concentration of ≥ 10^4^ colony-forming units/mL. The collection date of the index culture study was defined as the pneumonia index date.

### Data collection and definitions

Demographic characteristics and underlying comorbidities were obtained from hospital chart reviews. The disease severities were evaluated using the Acute Physiology and Chronic Health Evaluation (APACHE) II score on the day of ICU admission, Sequential Organ Failure Assessment (SOFA) scores on the pneumonia index date, and the presence of organ dysfunction upon pneumonia diagnosis, including septic shock (vasopressor use), renal failure (under dialysis), and respiratory failure (with mechanical ventilator and PaO2/FiO2 ratio < 200).

### Microbiological tests and treatment regimens

The results of susceptibility tests to carbapenems from the cultured isolates were determined based on the Clinical and Laboratory Standards Institute recommendations [21]. The isolates were considered susceptible to colistin if the minimum inhibitory concentrations were ≤ 2 mg/L in the broth microdilution method.

All the patients were not treated with intravenous colistin. Nebulized colistin administered during the treatment course of nosocomial pneumonia with a duration ≥ 2 days were recorded. Substitutive nebulized colistin was defined as nebulized colistin in conjunction with non-colistin intravenous antibiotics. The daily dose of substitutive nebulized colistin ranged from 2 million IU (MIU) colistimethate sodium (CMS) (66.8 mg colistin) to 15 MIU CMS (501 mg colistin) given 2 or 3 times a day. For dosage strategy analysis, we defined low-dose nebulized colistin as a daily dose ≤ 6 MIU CMS (200.4 mg colistin), and high-dose nebulized colistin as > 6 MIU CMS. Details of nebulized colistin preparation are shown in materials and methods of Additional file 1.

Intravenous antibiotics administered within 7 days of the pneumonia index date with ≥ 2-day duration were recorded. Inappropriate empirical therapy was considered when the patient did not receive at least one in vitro active antimicrobial agent within 24 h of pneumonia onset [22]. Meropenem was considered an active agent if it was prescribed in a dosage of 6 g/day in isolates with MIC ≤ 8 mg/L.

VAP prevention bundle, including hand hygiene, oral hygiene, daily sedation interruption, and head-of-bed elevation, is widely adopted in all the ICUs in the study hospital.

### Outcome evaluation

The primary endpoint was clinical responses on days 7, 14, and 28. Clinical responses were classified as cure (resolution of symptoms/signs of pneumonia, improvement or lack of progression of chest radiographic abnormalities, and antibiotics-free), improvement (substantial improvement of symptoms/signs of pneumonia, improvement or lack of progression of chest radiographic abnormalities, but remaining on antibiotic therapy), and failure (no apparent response to therapy, persistent or worsening of symptoms/signs of pneumonia, persistent or progression of radiographic abnormalities that required additional antibiotic therapy, or death) [23]. The secondary endpoints included all-cause mortality at day-14 and day-28, ventilator weaning rate at day-28, and new-onset dialysis at day-28. Details of clinical outcomes evaluation are provided in materials and methods of Additional file 1.

### Time window bias adjustment and propensity score matching

We created a second cohort for the adjustment of time-window bias related to delayed initiation of nebulized colistin [24]. Patients who died within 3 days of the onset of pneumonia, or who started nebulized colistin more than 3 days of the pneumonia index date, were excluded. After time-window bias adjustment, a propensity score (PS)-matched cohort was built using a PS approach with 1:1 matching and caliper width of 0.2 applied to both patients with and without nebulized colistin [25]. PS were created using logistic regression as a function of age, sex, smoking, pathogens, pneumonia types, ICU types, comorbidities, APACHE II scores, SOFA scores, albumin levels, organ failure, and key intravenous antibiotic used against CR-GNB. A PS-matched cohort was also made between patients with high- and low-dose nebulized colistin for sub-group analysis.

### Statistical analysis

Statistical analyses were performed using the SPSS version 25.0 software (SPSS, Inc., Chicago, IL, USA). Continuous variables were compared by the Mann–Whitney *U* test, and categorical variables with Pearson’s chi-square or Fisher’s exact tests, as appropriate. We used mean imputation for missing data.

Regarding treatment outcomes, clinical failure rate, all-cause mortality, ventilator weaning, and new-onset dialysis, were compared between patients with and without substitutive nebulized colistin; and patients with high- and low-dose nebulized colistin, both in original cohort and PS-matched cohort with time-window bias adjustment. Binary logistic regression analysis with stepwise selection was performed to determine the independent variables associated with clinical failure. All variables with *p* value < 0.1 in the univariate analysis were included in the multivariate model. All tests were two-tailed and a *p* value < 0.05 was considered statistically significant.

## Results

### Patients characteristics

**Fig. 1:**
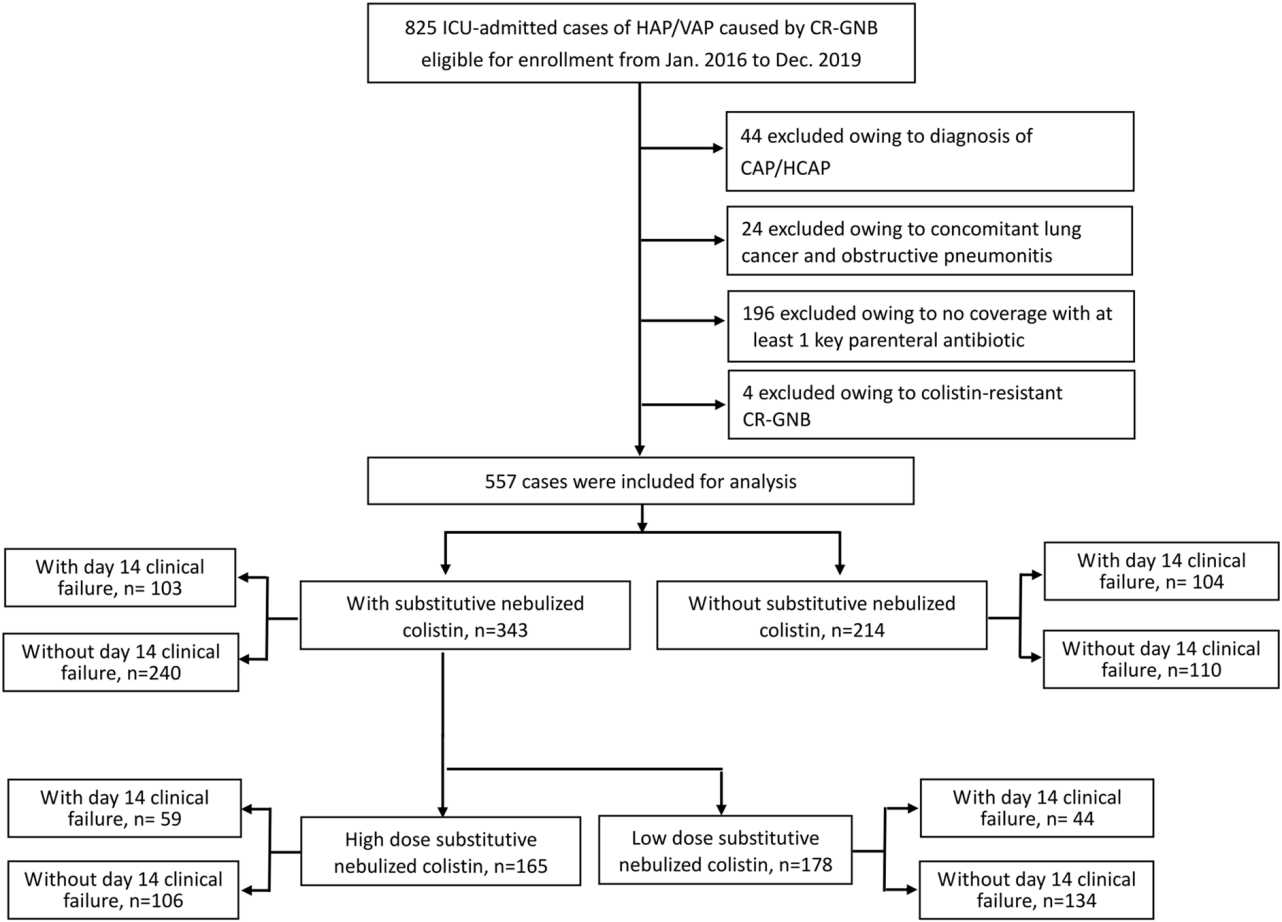
Study flow diagram and patient exclusion criteria. Low dose nebulized colistin was defined as a daily dose ≤ 6 MIU CMS, and high-dose nebulized colistin was defined as > 6 MIU CMS. CR-GNB, carbapenem-resistant Gram-negative bacteria; HAP, hospital acquired pneumonia; VAP, ventilator-associated pneumonia; ICU, intensive care unit

A total of 825 ICU-admitted patients with nosocomial pneumonia caused by CR-GNB were eligible for enrollment. A flow diagram showing the numbers of cases and reasons for exclusion is shown in Fig. 1. We included 557 cases, including 343 with and 214 without substitutive nebulized colistin, in the study. As summarized in Table 1, nosocomial pneumonia was mainly HAP (70.9%), caused by CRAB (81.5%), and 55.3% of the cases were admitted to medical ICU. The median APACHE II score upon ICU admission was 20 (interquartile range [IQR]: 16–24). During the onset of nosocomial pneumonia, the median SOFA score was 7 (IQR: 5–9), 13.1% had septic shock, and 21.4% had PF ratio < 200.

**Table 1:** Demographic characteristics and disease severities of ICU patients with nosocomial pneumonia treated with and without nebulized colistin in substitutive strategy^a^

	All cases	Substitutive nebulized colistin		*p* value
		Yes	No	
Case number	557	343	214	
Mean age (SD)	72.7 (16.0)	74.1 (15.6)	69.3 (16.3)	< 0.001
Male	391 (70.2%)	238 (69.4%)	153 (71.5%)	0.597
Mean BMI (SD)	23.0 (4.6)	23.0 (5.0)	23.1 (4.1)	0.670
Smoking history	172 (30.9%)	81 (23.6%)	91 (42.5%)	< 0.001
Isolated pathogens				< 0.001
CRAB	454 (81.5%)	304 (88.6%)	150 (70.1%)	
CRE	48 (8.6%)	25 (7.3%)	23 (10.7%)	
CR-Pseudomonas	55 (9.9%)	14 (4.1%)	41 (19.2%)	
Pneumonia types				0.736
HAP	395 (70.9%)	245 (71.4%)	150 (70.1%)	
VAP	162 (29.1%)	98 (28.6%)	64 (29.9%)	
ICU types				< 0.001
Medical ICU	308 (55.3%)	213 (62.1%)	95 (44.4%)	
Surgical ICU	249 (44.7%)	130 (37.9%)	119 (55.6%)	
Comorbidities				
Malignancies	112 (20.1%)	78 (22.7%)	34 (15.9%)	0.050
Renal insufficiency	126 (22.6%)	103 (30.0%)	23 (10.7%)	< 0.001
Chronic lung diseases^b^	109 (19.6%)	75 (21.9%)	34 (15.9%)	0.084
Diabetes	192 (34.5%)	130 (37.9%)	62 (29.0%)	0.031
Autoimmune disease	22 (3.9%)	19 (5.5%)	3 (1.4%)	0.015
Intravenous antibiotics				
Sulbactam	196 (35.2%)	136 (39.7%)	60 (28.0%)	0.005
Carbapenem	276 (49.6%)	135 (39.4%)	141 (65.9%)	< 0.001
Tigecycline	156 (28.0%)	110 (32.1%)	46 (21.5%)	0.007
Inappropriate empiric therapy	429 (77.0%)	260 (75.8%)	169 (79.0%)	0.387
APACHE II scores (Median, IQR)	20 (16–24)	20 (16–23)	22 (17–25)	< 0.001
SOFA scores (Median, IQR)	7 (5–9)	7 (5–9)	7 (4.8–9)	0.802
Presenting features^c^				
Septic shock	73 (13.1%)	48 (14.0%)	25 (11.7%)	0.432
Invasive ventilator	475 (85.3%)	281 (81.9%)	194 (90.7%)	0.005
PF ratio < 200	119 (21.4%)	74 (21.6%)	45 (21%)	0.878
Dialysis^d^	76 (13.6%)	61 (17.8%)	15 (7.0%)	< 0.001
Laboratory results (Median, IQR)				
Leukocytes (× 10^9^ per L)	10,900 (7685–15,100)	10,700 (7700–14,700)	11,265 (7637–16,057)	0.203
Albumin (g/dL)	3.0 (2.6–3.3)	3.1 (2.7–3.3)	2.8 (2.4–3.2)	< 0.001
CRP (mg/dL)	7.8 (3.3–12.4)	7.4 (3.2–11.4)	8.6 (3.9–13.7)	0.029
Nebulized colistin (Median, IQR)				
Dosage (MIU in CMS)	–	6 (4–15)	–	
Treatment duration (days)		7 (6–11)	–	

APACHE II, Acute Physiology and Chronic Health Evaluation II; BMI, body mass index; CR-Pseudomonas, carbapenem-*resistant Pseudomonas aeruginosa*; CRAB, carbapenem-resistant *Acinetobacter baumannii*; CRE, carbapenem-resistant Enterobacteriaceae; HAP, hospital acquired pneumonia; ICU, intensive care unit; IQR, interquartile range; PF ratio, PaO_2_/FiO_2_ ratio; SD, standard deviation; SOFA, Sequential Organ Failure Assessment; VAP, ventilator-associated pneumonia

^a^Data are presented as *n* (%)

^b^Including COPD, asthma, bronchiectasis, and pulmonary fibrosis

^c^Presence of organ dysfunction on pneumonia index date

^d^Including hemodialysis and continuous venovenous hemofiltration

Compared to patients without nebulized colistin, those with nebulized colistin were older, more likely to smoke, and be infected by CRAB. They also had medical ICU admission, renal insufficiency, diabetes, autoimmune diseases, lower APACHE II score upon ICU admission, dialysis after developing pneumonia, higher serum albumin level, lower CRP level, and were less likely to have a PF ratio < 200. Patients with nebulized colistin were more likely to receive intravenous sulbactam and tigecycline, but were less likely to be treated with carbapenem. The median duration of nebulized colistin treatment was 7 days (IQR: 6–14 days) and the median daily dose was 6 MIU CMS (IQR 4–15 MIU).

#### PS-matched cohort after time-window bias adjustment

After time-window bias adjustment and PS matching, we built a PS-matched cohort that included 115 patient pairs. As shown in Table 2, there were no significant differences in demographics, underlying comorbidities, disease severity, antibiotics used, or laboratory results between patients stratified according to nebulized colistin treatment in the PS-matched cohort.

**Table 2:** Demographic characteristics and disease severities of PS-matched ICU patients with nosocomial pneumonia treated with and without nebulized colistin in substitutive strategy^a^

	Substitutive nebulized colistin		*p* value
	Yes	No	
Case number	115	115	
Mean age (SD)	74.2 (15.5)	72.7 (15.6)	0.507
Male	83 (72.2%)	79 (68.7%)	0.563
Mean BMI (SD)	23.2 (5.0)	22.8 (4.4)	0.572
Smoking history	37 (32.2%)	41 (35.7%)	0.577
Isolated pathogens			0.636
CRAB	89 (77.4%)	94 (81.7%)	
CRE	13 (11.3%)	12 (10.4%)	
CR-Pseudomonas	13 (11.3%)	9 (7.8%)	
Pneumonia types			0.781
HAP	77 (67.0%)	75 (65.2%)	
VAP	38 (33.0%)	40 (34.8%)	
ICU types			0.787
Medical ICU	44 (38.3%)	46 (40.0%)	
Surgical ICU	71 (61.7%)	69 (60.0%)	
Comorbidities			
Malignancies	18 (15.7%)	18 (15.7%)	1.000
Renal insufficiency	24 (20.9%)	18 (15.7%)	0.306
Chronic lung diseases^b^	24 (20.9%)	20 (17.4%)	0.502
Diabetes	40 (34.8%)	37 (32.2%)	0.675
Autoimmune disease	5 (4.3%)	2 (1.7%)	0.250
Intravenous antibiotics			
Sulbactam	37 (32.2%)	43 (37.4%)	0.406
Carbapenem	52 (45.2%)	52 (45.2%)	1.000
Tigecycline	29 (25.2%)	30 (26.1%)	0.880
Inappropriate empiric therapy	88 (76.5%)	87 (75.7%)	0.877
APACHE II scores (Median, IQR)	21 (17–24)	21 (16–24)	0.937
SOFA scores (Median, IQR)	7 (5–8)	6 (4–9)	0.548
Presenting features^c^			
Septic shock	8 (7.0%)	11 (9.6%)	0.472
Invasive ventilator	97 (84.3%)	102 (88.7%)	0.334
PF ratio < 200	28 (24.3%)	22 (19.1%)	0.337
Dialysis^d^	15 (13.0%)	7 (6.1%)	0.073
Laboratory results (median, IQR)			
Leukocytes (× 10^9^ per L)	11,100 (7600–14,900)	11,600 (7900–15,955)	0.420
Albumin (g/dL)	3.0 (2.6–3.3)	2.8 (2.5–3.2)	0.188
CRP (mg/dL)	7.6 (3.3–11.2)	8.0 (3.4–12.3)	0.246
Nebulized colistin (Median, IQR)			
Dosage (MIU in CMS)	6 (4–15)	-	
Treatment duration (days)	7 (5–13)	-	

APACHE II, Acute Physiology and Chronic Health Evaluation II; BMI, body mass index; CR-Pseudomonas, carbapenem-*resistant Pseudomonas aeruginosa*; CRAB, carbapenem-resistant *Acinetobacter baumannii*; CRE, carbapenem-resistant Enterobacteriaceae; HAP, hospital acquired pneumonia; ICU, intensive care unit; IQR, interquartile range; PF ratio, PaO_2_/FiO_2_ ratio; SD, standard deviation; SOFA, Sequential Organ Failure Assessment; VAP, ventilator-associated pneumonia

^a^Data are presented as n (%)

^b^Including COPD, asthma, bronchiectasis, and pulmonary fibrosis

^c^Presence of organ dysfunction on pneumonia index date

^d^Including hemodialysis and continuous venovenous hemofiltration

#### Substitutive nebulized colistin was associated with a better treatment outcome

As shown in Table 3, in the original cohort, patients with substitutive nebulized colistin had lower clinical failure rates on days 7, 14, and 28, lower mortality rates on days 14 and 28, as well as longer ICU and hospital stays. In PS-matched cohort after survival time bias adjustment, clinical failure rate on days 7 (22.6 vs. 42.6%, *p* = 0.001), 14 (27 vs. 42.6%, *p* = 0.013), and 28 (27.8 vs. 41.7%, *p* = 0.027) was significantly lower in patients with substitutive nebulized colistin. The mortality rate, ventilator weaning rate, and occurrence of new-onset dialysis were comparable between patients with and without substitutive nebulized colistin.

**Table 3:** Treatment outcomes of ICU patients with nosocomial pneumonia treated with and without nebulized colistin in substitutive strategy^a^

	Original cohort		*p* value	PS-matched cohort		*p* value
	With substitutive nebulized colistin	Without substitutive nebulized colistin		With substitutive nebulized colistin	Without substitutive nebulized colistin	
Case number	343	214		115	115	
Clinical failure						
Day 7	95 (27.7%)	102 (47.7%)	< 0.001	26 (22.6%)	49 (42.6%)	0.001
Day 14	103 (30.0%)	104 (48.6%)	< 0.001	31 (27.0%)	49 (42.6%)	0.013
Day 28	102 (29.7%)	104 (48.6%)	< 0.001	32 (27.8%)	48 (41.7%)	0.027
All-cause mortality						
Day 14	48 (14.0%)	56 (26.2%)	< 0.001	12 (10.4%)	21 (18.3%)	0.090
Day 28	78 (22.7%)	67 (31.3%)	0.025	19 (16.5%)	26 (22.6%)	0.245
Hospital mortality	136 (39.7%)	97 (45.3%)	0.186	42 (36.5%)	42 (36.5%)	1.000
28-day ventilator weaning^b^	164 (55.8%)	96 (48.5%)	0.112	55 (55.6%)	59 (55.7%)	0.988
28-day new dialysis	20 (5.8%)	12 (5.6%)	0.912	8 (7.0%)	9 (7.8%)	0.801
ICU stays (Median, IQR)	32.1 (24.2)	26.9 (22.1)	0.011	25 (15–47)	23 (14–40)	0.245
Hospital stays (Median, IQR)	63.5 (45.1)	51.0 (36.2)	0.001	53 (34–90)	48 (31–71)	0.332

^a^Data are presented as n (%)

^b^Only cases with invasive ventilator were included for analysis

**Fig. 2:**
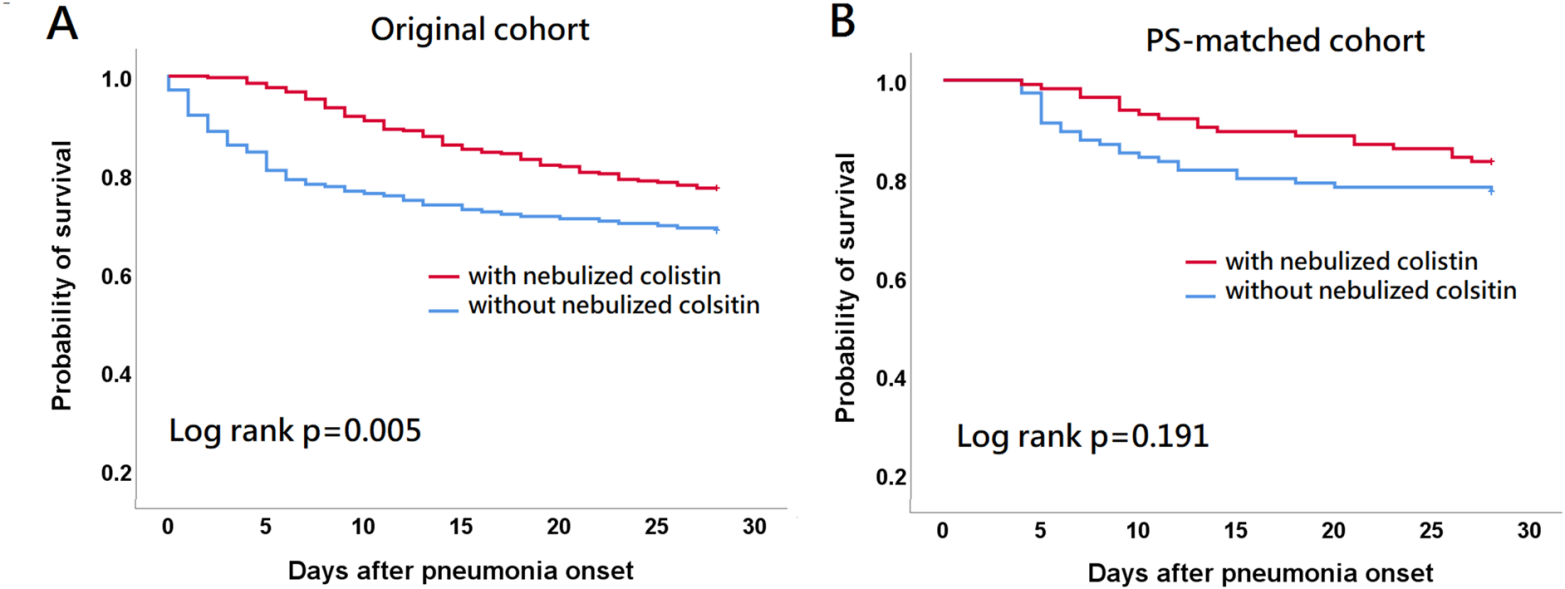
Kaplan–Meier analysis of 28-day mortality status. 28-day mortality between patients with and without substitutive nebulized colistin in (**A**) original and (**B**) propensity score-matched cohorts is compared

Kaplan–Meier analyses of all-cause mortalities in the original and PS-matched cohorts are shown in Fig. 2. In the original cohort, patients with substitutive nebulized colistin had a significantly lower 28-day mortality risk compared to patients without substitutive nebulized colistin (log rank *p* = 0.005). The curves separated early after pneumonia onset. In the PS-matched cohort, there was no significant difference in 28-day mortality risk between patients with and without substitutive nebulized colistin.

Microbiological eradication rates on day 14, and day 28 were significantly higher in patients with nebulized colistin in both the original and PS-matched cohorts. (Additional file 1: Table S1).

#### Independent factors associated with treatment outcomes

Univariate and multivariate analyses were performed to identify independent clinical factors associated with clinical failure at day 14. As shown in Table 4, in the original cohort, independent factors associated with clinical failure on day 14 included medical ICU admission (adjusted odds ratio [aOR]: 1.58, 95% confidence interval [CI] 1.06–2.35), SOFA scores at pneumonia onset (aOR: 1.12, 95% CI 1.05–1.19), PF ratio ≤ 200 (aOR: 1.83, 95% CI 1.13–2.94), inappropriate empirical therapy (aOR: 1.64, 95% CI 1.04–2.59), and substitutive nebulized colistin (aOR: 0.45, 95% CI 0.30–0.67). In the PS-matched cohort, independent factors associated with clinical failure on day 14 included SOFA scores on the pneumonia index date (aOR: 1.16, 95% CI 1.05–1.28), and substitutive nebulized colistin (aOR: 0.48, 95% CI 0.27–0.87).

**Table 4:** Univariate and multivariate analyses of clinical factors associated with day 14 clinical failure in ICU patients with nosocomial pneumonia caused by CR-GNB

	Original cohort^a^				PS-matched cohort^a^			
	Univariate analysis		Multivariate analysis		Univariate analysis		Multivariate analysis	
	OR (95% CI)	*p* value	aOR (95% CI)	*p* value	OR (95% CI)	*p* value	aOR (95% CI)	*p* value
Age	1.00 (0.99–1.01)	0.554			0.98 (0.97–1)	0.041	0.99 (0.97–1.01)	0.175
Male	1.15 (0.79–1.67)	0.479			0.97 (0.54–1.75)	0.916		
BMI	0.99 (0.96–1.03)	0.674			0.96 (0.91–1.02)	0.211		
CRAB	1.12 (0.72–1.76)	0.607			0.93 (0.48–1.81)	0.823		
Medical ICU	1.53 (1.08–2.17)	0.017	1.58 (1.06–2.35)	0.024	0.9 (0.52–1.57)	0.711		
Smoking	1.12 (0.73–1.71)	0.615			0.99 (0.56–1.76)	0.970		
Malignancies	0.85 (0.52–1.39)	0.519			1.07 (0.51–2.25)	0.855		
Renal insufficiency	0.93 (0.60–1.43)	0.727			1.72 (0.87–3.4)	0.118		
Chronic lung diseases	0.70 (0.48–1.01)	0.054	1.07 (0.66–1.72)	0.790	1.09 (0.55–2.16)	0.807		
Diabetes	1.05 (1.02–1.08)	< 0.001	0.76 (0.51–1.14)	0.185	0.66 (0.36–1.19)	0.162		
APACHE II score	1.17 (1.11–1.23)	< 0.001	1.03 (1.00–1.06)	0.056	1.01 (0.96–1.05)	0.769		
SOFA score^c^	2.51 (1.66–3.79)	< 0.001	1.12 (1.05–1.19)	0.001	1.19 (1.09–1.29)	0.000	1.16 (1.05–1.28)	0.004
PF ratio ≤ 200^c^	2.30 (1.40–3.78)	0.001	1.83 (1.13–2.94)	0.013	2.27 (1.2–4.3)	0.012	1.57 (0.74–3.33)	0.242
Septic shock ^c^	1.19 (0.73–1.96)	0.482			1.78 (0.69–4.57)	0.234		
Albumin ≤ 3 mg/dL	1.48 (1.05–2.09)	0.025	1.4 (0.95–2.05)	0.088	1.39 (0.81–2.4)	0.234		
Inappropriate empiric therapy	1.54 (1.01–2.36)	0.047	1.64 (1.04–2.59)	0.033	1.77 (0.90–3.50)	0.098	2.01 (0.98–4.14)	0.058
Substitutive nebulized colistin^d^	0.45 (0.32–0.65)	< 0.001	0.45 (0.30–0.67)	< 0.001	0.5 (0.29–0.87)	0.013	0.48 (0.27–0.87)	0.015

APACHE II, Acute Physiology and Chronic Health Evaluation II; CRAB, carbapenem-resistant *Acinetobacter baumannii*; ICU, intensive care unit; PF ratio, PaO_2_/FiO_2_ ratio; SOFA, Sequential Organ Failure Assessment

^a^Adjusted odds ratio (aOR) and 95%CI were derived from logistic regression analysis

^b^SOFA score on pneumonia index date

^c^Presence of organ dysfunction on the pneumonia index date

^d^As substitutive strategy

#### Impact of dosage of substitutive nebulized colistin on treatment outcomes

We performed sub-group analysis to investigate the impact of nebulized colistin dosage in treatment outcomes. The demographics of patients with high- (> 6 MIU/day) and low-dose (≤ 6 MIU/day) nebulized colistin in the original and PS-matched cohorts is shown in Additional file 1: Table S2. In the original cohort, as compared to patients with high dose nebulized colistin, those with low doses were more likely to be male, have HAP, have diabetes, and were less likely to be treated with carbapenem and tigecycline. The median daily dosage of nebulized colistin were 4 MIU and 15 MIU, and median treatment duration were 7 and 8 days, in patients with low and high dose nebulized colistin, respectively. In the PS-matched cohort with 124 patient pairs, there were no significant differences in demographics between patients with low- and high-dose nebulized colistin.

Comparisons of treatment outcomes between patients with low- and high-dose nebulized colistin are shown in Additional file 1: Table S3. In the original cohort, patients with high-dose nebulized colistin had higher clinical failure rate on days 7, 14, and 28, as well as higher 28 days and hospital mortalities. In the PS-matched cohort, there were no differences in clinical failure and mortality rates between patients with low- and high-dose nebulized colistin. The 28-day ventilator weaning and occurrence rates of 28-day new-onset dialysis were also comparable between the two groups.

Kaplan–Meier analyses of all-cause mortalities between patients with high and low dose nebulized colistin in the original and PS-matched cohorts are shown in Additional file 1: Figure S1. In the original cohort, patients with low dose nebulized colistin had significantly lower 28-day mortality risk compared to patients with high dose nebulized colistin (log rank *p* = 0.004). In the PS-matched cohort, there was no significant difference in 28-day mortality risk between patients with high and low dose nebulized colistin.

**Fig. 3:**
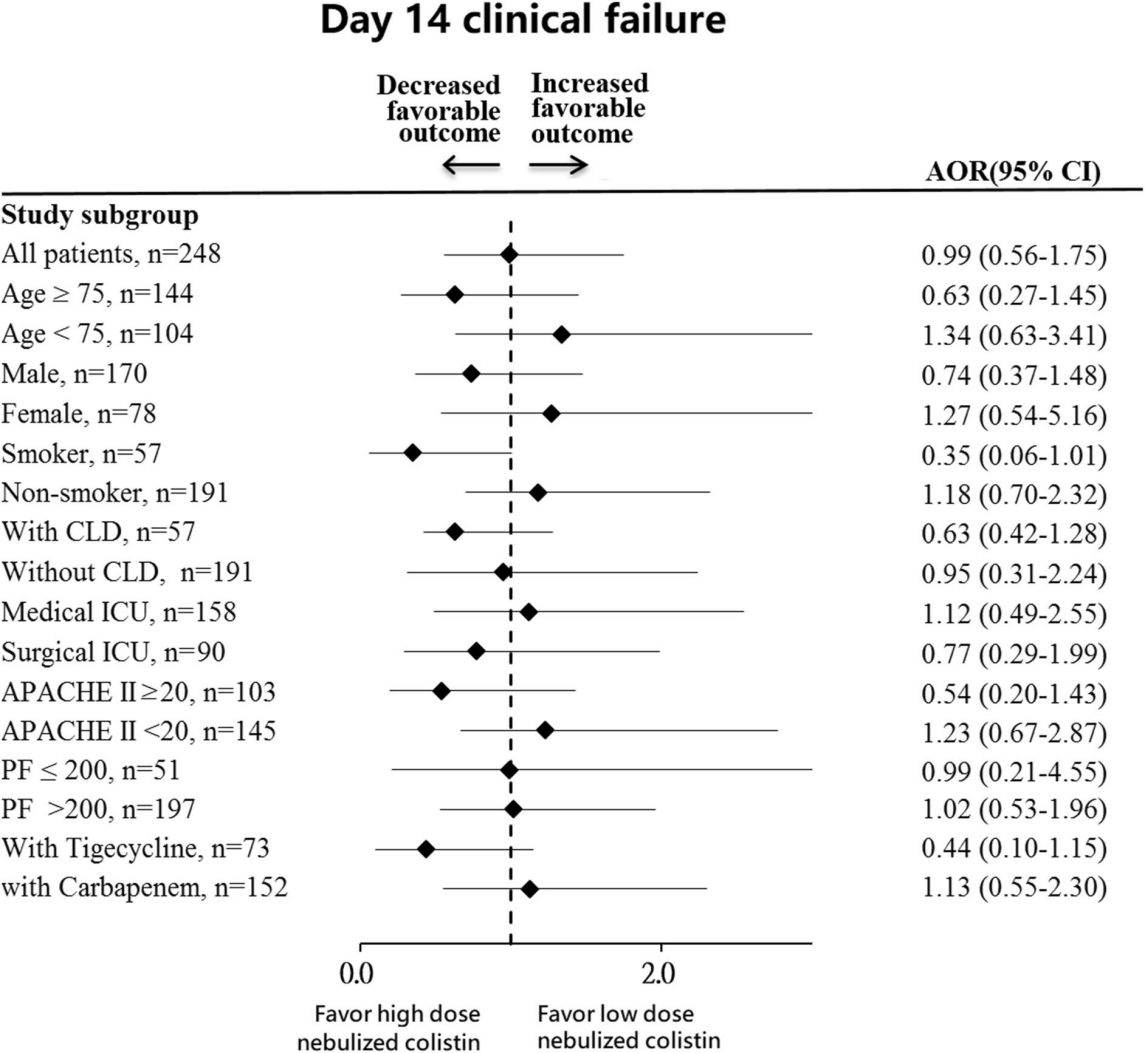
Forest plot analysis for the association between substitutive nebulized colistin dosage and day-14 clinical failure. ICU-admitted patients with nosocomial pneumonia caused by CR-GNB were categorized as high-dose nebulized colistin (> 6 MIU/day CMS) and low-dose nebulized colistin (≤ 6 MIU/day CMS). ICU, intensive care units; CR-GNB, carbapenem-resistant Gram-negative bacteria; CLD, chronic lung disorders (including COPD, asthma, bronchiectasis, and pulmonary fibrosis); VAP, ventilator-associated pneumonia; HAP, hospital-acquired pneumonia; APACHE II, Acute Physiology and Chronic Health Evaluation II; PF, PaO_2_/FiO_2_ ratio

Sub-group analysis was performed to identify specific populations that may benefit from high-dose nebulized colistin in the PS-matched cohort. As shown in Fig. 3, no significant differences in clinical failure rate were found in all the sub-group of patients. However, patients with age ≥ 75 years, smoker, with chronic lung disease, with APACHE II score ≥ 20, and with tigecycline treatment, may benefit more from high-dose nebulized colistin with a trend of lower clinical failure rate.

## Discussion

This retrospective cohort study included critically ill patients with HAP/VAP caused by CR-GNB and investigated the clinical efficacy of substitutive nebulized colistin. In the multivariate analysis in the PS-matched cohort, we found that substitutive nebulized colistin is an independent factor associated with fewer cases of day 14 clinical failure. Meanwhile, substitutive nebulized colistin was not associated with new-onset dialysis. We also found that patients treated with high- and low-dose substitutive nebulized colistin had similar treatment outcomes, including clinical failure and all-cause mortality rates.

Colistin is widely used as one of the key agents against CR-GNB. Adverse reactions, especially nephrotoxicity, and low concentrations in lung tissue seriously limited its use for critically ill patients with HAP or VAP [16, 17, 26]. Colistin is a concentration-dependent antibiotic with a post-antibiotic effect, which makes it particularly suitable for nebulization as high lung concentrations can be expected [27]. Adjunctive nebulized colistin has been shown to improve clinical and microbiological responses in patients with pneumonia caused by CR-GNB [28–31]. However, due to insufficient randomized controlled trials, the role of adjunctive nebulized colistin in pneumonia caused by CR-GNB remains controversial in the latest treatment guidelines [4, 11–13, 19].

Substitutive nebulized colistin refers to nebulized colistin without concomitant intravenous colistin. It is sometimes prescribed in patients with renal insufficiency or when nephrotoxicity is a concern, especially in ICU-admitted patients. Studies related to substitutive nebulized colistin is very limited. A randomized, single-blind study compared the efficacy of substitutive nebulized colistin with intravenous colistin in patients with community acquired pneumonia caused by multidrug-resistant GNB [32]. They reported that substitutive nebulized colistin had comparable treatment effects with intravenous colistin and had a lower risk of nephrotoxicity. Kuo et al. conducted a small retrospective study on patients with pneumonia caused by multidrug-resistant *Acinetobacter baumannii* (MDRAB) [33]. They found that substitutive nebulized colistin was significantly associated with MDRAB eradication as compared with matched control that received non-colistin antibiotics [33]. In this retrospective cohort study, we included more than 500 patients with HAP/VAP caused by CR-GNB, and created a PS-matched cohort that included 115 patient pairs. We found that patients given substitutive nebulized colistin were associated with less clinical failure as compared to those without substitutive nebulized colistin, although the mortality rate were comparable between the two groups of patients. We also reported similar incidence rates of new onset dialysis, which, again, demonstrated the low risk of nephrotoxicity in nebulized colistin.

The optimal daily dose and treatment duration for nebulized colistin remains unclear. The mean daily dose of nebulized colistin ranged from 1.25 to 15 MIU and the mean duration of treatment ranged from 7 to 17.5 days in previous studies [32–36]. Due to concerns for heterogeneously distributed inhaled antibiotics in lung tissues and possible low drug deposition in the affected lobe [37], we speculated that patients with pneumonia may benefit more from higher doses of nebulized antibiotics. However, there were no direct comparisons regarding treatment outcomes between different dosing strategies of nebulized colistin to date. To the best of our knowledge, this is the first study evaluating the impact of nebulized colistin dosage in treatment outcomes of pneumonia. Possibly due to more comorbidities and more organ dysfunction in high dose group, in the original cohort, patients treated with high daily doses nebulized colistin had worse treatment outcomes. After PS matching, patients treated with high and low daily doses had comparable treatment outcomes. Interestingly, we found that patients with older age, smoking habit, chronic lung diseases, lower PF ratio, and concomitant tigecycline treatment, may have less clinical failure when using high dose substitutive nebulized colistin. Although the evidences remain weak, our findings suggested that the dosing strategy of nebulized colistin may affect treatment outcomes in specific populations of patients with nosocomial pneumonia.

This study had several limitations. First, as a retrospective cohort study, there are marked differences in the demographic characteristics and disease severities. The survival time bias related to late initiation of nebulized colistin may overestimate its benefit in mortality rate. However, we have established a PS-matched cohort after adjusting survival time bias to minimize confounders in our analysis. Second, there is no standardized protocol for nebulized colistin, including dosage and duration, in our enrolled patients [38]. However, it provided us the opportunities to elucidate the dosage issues of nebulized colistin dosage on treatment outcomes of these critically ill patients. Third, nebulized colistin was performed with jet nebulizer, not mesh nebulizer. Jet nebulizer may lead to lower lung deposition of colistin and underestimate the clinical efficacy of nebulized colistin [39]. However, all the enrolled cases had jet nebulizers in the standard procedure in this single center study. Finally, this study was conducted in a tertiary medical center specialized in critical care, and only HAP/VAP caused by CR-GNB were included for analysis. Therefore, caution should be exercised in applying our present findings in other clinical settings with non-CR-GNB pathogens.

## Conclusions

This retrospective cohort study included critically ill patients with HAP/VAP caused by CR-GNB. A majority of the patients were infected by CRAB. As compared to patients treated with non-colistin regimen, patients treated with substitutive nebulized colistin had lower clinical failure rate and similar mortality rate. We also found that patients treated with high- and low-dose nebulized colistin had comparable treatment outcomes and nephrotoxicity risk. Sub-group analysis suggested that patients with older age, with smoking habits, with chronic lung disorders, and with higher severities may benefit more from high-dose nebulized colistin. Our findings are only applicable in settings with endemic HAP/VAP caused by CR-GNB, mainly CRAB. Further prospective study is deserved to elucidate the clinical benefits of substitutive nebulized colistin, including the application of high dose nebulized colistin in specific population.

## Supplementary Information

**Additional file 1.** Materials and methods.

## Data Availability

The data sets generated during and/or analysed during the current study are available from the corresponding author on reasonable request.

## Abbreviations

HAP: Hospital-acquired pneumonia
VAP: Ventilator-associated pneumonia
ICU: Intensive care unit
CR-GNB: Carbapenem-resistant Gram-negative bacteria
CRAB: Carbapenem-resistant *Acinetobacter baumannii* complex
CRE: Carbapenem-resistant enterobacteriaceae
CRPA: CR-*Pseudomonas aeruginosa*
APACHE: Acute Physiology and Chronic Health Evaluation
SOFA: Sequential Organ Failure Assessment
CMS: Colistimethate sodium
PS: Propensity score
aOR: Adjusted odds ratio
CI: Confidence interval
