# Supplementary material for: Role of nebulized colistin as a substitutive strategy against nosocomial pneumonia caused by CR-GNB in intensive care units: a retrospective cohort study
Source: Ann Intensive Care. 2023 Jan 7;13:1. doi: 10.1186/s13613-022-01088-4 (PMC9825688; doi:10.1186/s13613-022-01088-4)

**Supplementary materials**

**Materials and methods**

*Outcome evaluation*

Clinical responses were evaluated on day 7, 14, and 28, and were classified as clinical cure, clinical improvement, and clinical failure. Patients were considered clinical cure if the following criteria were met: resolution of baseline signs/symptoms of pneumonia (leukocytosis, fever, purulent sputum production), a remarkable improvement in PF ratio, improvement or lack of progression of chest radiographic abnormalities, and free of antibiotics. Patients were considered clinical improvement if the following criteria were met: substantial improvement of baseline signs/symptoms of pneumonia, improvement in PF ratio, improvement or lack of progression of chest radiographic abnormalities, and remaining on antibiotic therapy for pneumonia. Patients were considered clinical failure if the following criteria were met: no apparent response to therapy, persistent or worsening of signs/symptoms of pneumonia, no improvement PF ratio, progression of radiographic abnormalities that required additional antibiotic therapy, or death. The clinical outcomes were reviewed by an independent reviewer who was blinded to the group. Clinical information used to determine clinical outcomes was obtained from electric medical records in Taipei Veterans General Hospital.

*Nebulized colistin preparation*

Nebulized colistin was administered with a jet nebulizer in all the enrolled patients. Nebulized colistin was

prepared by mixing colistimethate sodium with 6~10 mL of normal saline immediately before nebulization. In patients with mechanical ventilator, a jet nebulizer was placed at 15 cm upstream of the Y-piece and connected to the inspiratory outlet of the ventilator. During the nebulization period, volume-controlled mode with constant inspiratory flow was suggested. Settings of tidal volume 8ml/kg and respiratory rate 12/min were recommended and conventional humidifier was off. Light sedation was used in patients with agitation and ventilator asynchrony, but was not routinely required.

Supplementary Table 1. Treatment outcomes of ICU patients with nosocomial pneumonia treated with and without nebulized colistin in substitutive strategy

|  | Original Cohort | |  | PS-matched cohort | |  |
| --- | --- | --- | --- | --- | --- | --- |
|  | With substitutive nebulized colistin | Without substitutive nebulized colistin | *P* value | With substitutive nebulized colistin | Without substitutive nebulized colistin | *P* value |
| **Case number** | 343 | 214 |  | 115 | 115 |  |
| Microbiological eradication^a^ |  |  |  |  |  |  |
| Day 7 | 72 (42.4%) | 27 (28.7%) | 0.028 | 24 (38.7%) | 16 (28.6%) | 0.256 |
| Day 14 | 179 (71.0%) | 43 (28.7%) | <0.001 | 56 (69.1%) | 25 (35.7%) | <0.001 |
| Day 28 | 197 (79.1%) | 64 (51.2%) | <0.001 | 59 (71.1%) | 42 (51.9%) | 0.011 |

^a^Only cases with evaluable microbiological outcomes were included for analysis

**Supplementary Table 2.** Demographic characteristics and disease severities of ICU patients with nosocomial pneumonia treated with high-dose and low-dose substitutive nebulized colistin in substitutive strategy^a^

|  | Original cohort | | P value | PS-matched cohort | | P value |
| --- | --- | --- | --- | --- | --- | --- |
|  | Low dose substitutive nebulized colistin^b^ | High dose substitutive nebulized colistin^b^ |  | Low dose substitutive nebulized colistin^b^ | High dose substitutive nebulized colistin^b^ |  |
| Case number | 178 | 165 |  | 124 | 124 |  |
| Mean age (SD) | 73.3 (15.7) | 75.8 (15.4) | 0.312 | 75.0 (16.2) | 75.3 (15.0) | 0.864 |
| Male | 132 (74.2%) | 106 (64.2%) | 0.047 | 83 (66.9%) | 87 (70.2%) | 0.584 |
| Mean BMI (SD) | 23.4 (4.8) | 22.6 (4.9) | 0.217 | 22.6 (4.8) | 22.9 (5.0) | 0.659 |
| Smoking history | 39 (21.9%) | 42 (25.5%) | 0.440 | 27 (21.8%) | 30 (24.2%) | 0.651 |
| Isolated pathogens |  |  | 0.731 |  |  | 0.265 |
| CRAB | 158 (88.8%) | 146 (88.5%) |  | 108 (87.1%) | 112 (90.3%) |  |
| CRE | 14 (7.9%) | 11 (6.7%) |  | 11 (8.9%) | 5 (4.0%) |  |
| CR-Pseudomonas | 6 (3.4%) | 8 (4.8%) |  | 5 (4.0%) | 7 (5.6%) |  |
| Pneumonia types |  |  | 0.029 |  |  | 0.889 |
| HAP | 118 (66.3%) | 127 (77.0%) |  | 88 (71.0%) | 87 (70.2%) |  |
| VAP | 60 (33.7%) | 38 (23.0%) |  | 36 (29.0%) | 37 (29.8%) |  |
| ICU types |  |  | 0.905 |  |  | 1.000 |
| Medical ICU | 110 (61.8%) | 103 (62.4%) |  | 79 (63.7%) | 79 (63.7%) |  |
| Surgical ICU | 68 (38.2%) | 62 (37.6%) |  | 45 (36.3%) | 45 (36.3%) |  |
| Comorbidities |  |  |  |  |  |  |
| Malignancies | 34 (19.1%) | 44 (26.7%) | 0.095 | 28 (22.6%) | 30 (24.2%) | 0.764 |
| Renal insufficiency | 53 (29.8%) | 50 (30.3%) | 0.915 | 30 (24.2%) | 38 (30.6%) | 0.255 |
| Chronic lung diseases^c^ | 38 (21.3%) | 37 (22.4%) | 0.810 | 31 (25.0%) | 26 (21.0%) | 0.450 |
| Diabetes | 78 (43.8%) | 52 (31.5%) | 0.019 | 43 (34.7%) | 44 (35.5%) | 0.894 |
| Autoimmune disease | 7 (3.9%) | 12 (7.3%) | 0.177 | 7 (5.6%) | 7 (5.6%) | 1.000 |
| Intravenous antibiotics |  |  |  |  |  |  |
| Sulbactam | 67 (37.6%) | 69 (41.8%) | 0.429 | 53 (42.7%) | 52 (41.9%) | 0.898 |
| Carbapenem | 93 (52.2%) | 110 (66.7%) | 0.007 | 72 (58.1%) | 80 (64.5%) | 0.297 |
| Tigecycline | 46 (25.8%) | 64 (38.8%) | 0.010 | 39 (31.5%) | 34 (27.4%) | 0.486 |
| APACHE II scores (Median, IQR) | 20 (15-23) | 20 (36-24) | 0.519 | 20 (16-23) | 20 (16-23) | 0.462 |
| SOFA scores (Median, IQR) | 7 (5-9) | 7 (5-9) | 0.361 | 7 (5-9) | 7 (5-9) | 0.650 |
| Presenting features^d^ |  |  |  |  |  |  |
| Septic shock | 22 (12.4%) | 26 (15.8%) | 0.365 | 15 (12.1%) | 15 (12.1%) | 1.000 |
| Invasive ventilator | 140 (78.7%) | 141 (85.5%) | 0.102 | 98 (79.0%) | 104 (83.9%) | 0.327 |
| PF ratio <200 | 34 (19.1%) | 40 (24.2%) | 0.247 | 26 (21.0%) | 25 (20.2%) | 0.875 |
| Dialysis^e^ | 29 (16.3%) | 32 (19.4%) | 0.453 | 25 (20.2%) | 26 (21.0%) | 0.827 |
| Laboratory results (Median, IQR)^c^ |  |  |  |  |  |  |
| Leukocytes (x 10^9^ per L) | 10300 (7800-14800) | 10900 (7500-14350) | 0.915 | 10800 (7950-14750) | 10900 (7600-14250) | 0.849 |
| Albumin (g/dL) | 3.1 (2.7-3.3) | 3.1 (2.7-3.4) | 0.549 | 3.1 (2.8-3.4) | 3.1 (2.7-3.4) | 0.637 |
| CRP (mg/dL) | 7.2 (3.0-10.4) | 8.1 (3.3-13.0) | 0.516 | 7.1 (3.2-10.6 ) | 6.6 (2.8-11.6) | 0.856 |
| Nebulized colistin (Median, IQR) |  |  |  |  |  |  |
| Dosage (MIU/day, CMS) | 4 (4-4) | 15 (12-15) | <0.001 | 4 (4-4) | 15 (12-15) | <0.001 |
| Treatment duration (days) | 7 (5-10) | 8 (6-12) | 0.201 | 7 (5-10) | 8 (7-12) | 0.168 |

^a^Data are presented as n (%)

^b^high dose nebulized colistin (> 6MIU/day CMS) and low dose nebulized colistin (≤ 6 MIU/day CMS)

^c^Including COPD, asthma, bronchiectasis, and pulmonary fibrosis

^d^Presence of organ dysfunction on pneumonia index date

^e^Including hemodialysis and continuous venovenous hemofiltration

Abbreviations: APACHE II, Acute Physiology and Chronic Health Evaluation II; BMI, body mass index; CRP, carbapenem-*resistant Pseudomonas aeruginosa*; CRAB, carbapenem-resistant *Acinetobacter baumannii*; CRE, carbapenem-resistant Enterobacteriaceae; HAP, hospital acquired pneumonia; ICU, intensive care unit; IQR, interquartile range; PF ratio, PaO_2_/FiO_2_ ratio; SD, standard deviation; SOFA, Sequential Organ Failure Assessment; VAP, ventilator-associated pneumonia.

**Supplementary Table 3.** Treatment outcomes of ICU patients with nosocomial pneumonia treated with lower and higher doses of nebulized colistin as substitutive strategy^a^

|  | Original Cohort | |  | PS-matched cohort | |  |
| --- | --- | --- | --- | --- | --- | --- |
|  | Low dose substitutive nebulized colistin^b^ | High dose substitutive nebulized colistin^b^ | *P* value | Low dose substitutive nebulized colistin^b^ | High dose substitutive nebulized colistin^b^ | *P* value |
| **Case number** | 178 | 165 |  | 124 | 124 |  |
| Clinical failure |  |  |  |  |  |  |
| Day 7 | 40 (22.5%) | 55 (33.3%) | 0.025 | 33 (26.4%) | 39 (31.2%) | 0.402 |
| Day 14 | 44 (24.7%) | 59 (35.8%) | 0.026 | 36 (28.8%) | 40 (32.0%) | 0.582 |
| Day 28 | 42 (23.6%) | 60 (36.4%) | 0.010 | 36 (28.8%) | 42 (33.6%) | 0.413 |
| All-cause mortality |  |  |  |  |  |  |
| Day 14 | 20 (11.2%) | 28 (17.0%) | 0.126 | 16 (12.8%) | 18 (14.4%) | 0.712 |
| Day 28 | 29 (16.3%) | 49 (29.7%) | 0.003 | 24 (19.2%) | 31 (24.8%) | 0.285 |
| Hospital mortality | 58 (32.6%) | 78 (47.3%) | 0.005 | 50 (40.0%) | 53 (42.4%) | 0.700 |
| 28-day ventilator weaning^c^ | 90 (60.8%) | 74 (50.7%) | 0.080 | 58 (55.2%) | 55 (50.0%) | 0.442 |
| 28-day new dialysis | 11 (6.2%) | 9 (5.5%) | 0.775 | 9 (7.2%) | 4 (3.2%) | 0.108 |
| ICU stays (Median, IQR) | 23 (14-41) | 27 (16-43) | 0.095 | 23 (14-41) | 28 (16-43) | 0.219 |
| Hospital stays (Median, IQR) | 51 (33-83) | 51 (36-81) | 0.528 | 51 (33-90) | 58 (36-84) | 0.752 |

^a^Data are presented as n (%)

^b^high dose nebulized colistin (> 6MIU/day CMS) and low dose nebulized colistin (≤ 6 MIU/day CMS)

^c^Only cases with invasive ventilator were included for analysis

**Supplementary Figure 1.** Kaplan–Meier analysis of 28-day mortality status. 28-day mortality between patients with high- and low-dose substitutive nebulized colistin, in the A) original and B) propensity score-matched cohorts is compared. HD, high dose (> 6 MIU/day CMS); LD, low dose (≤ 6MIU/day CMS).


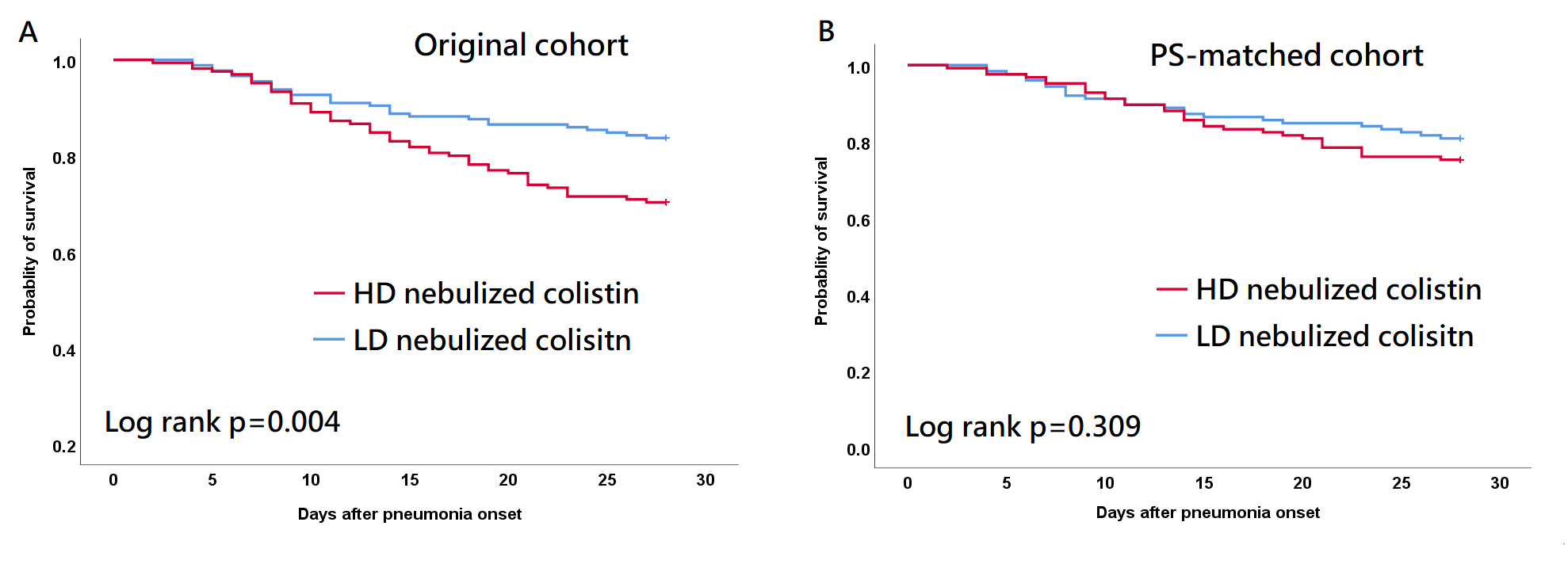

Supplement: Supplementary file 1 — Additional file 1. Materials and methods. [file 13613_2022_1088_MOESM1_ESM.doc]
